# Penta­zirconium copper tribismuth

**DOI:** 10.1107/S1600536813019235

**Published:** 2013-07-17

**Authors:** Agnieszka Balinska, Ivan Tarasiuk, Volodymyr Pavlyuk

**Affiliations:** a Institute of Chemistry, Environment Protection and Biotechnology, Jan Dlugosz University, al. Armii Krajowej 13/15, 42-200 Czestochowa, Poland; b Department of Inorganic Chemistry, Ivan Franko Lviv National University, Kyryla and Mefodiya str. 6, 79005, Lviv, Ukraine

## Abstract

Penta­zirconium copper tribismuth, Zr_5_CuBi_3_, crystallizes in the hexa­gonal Hf_5_CuSn_3_ structure type. The asymmetric unit contains two Zr sites (site symmetries 3.2 and *m*2*m*), one Cu site (site symmetry 3.*m*) and one Bi site (site symmetry *m*2*m*). The environment of the Bi atoms is a tetra­gonal anti­prism with one added atom and a coordination number (CN) of 9. The polyhedron around the Zr1 atom is a defective cubo­octa­hedron with CN = 11. The bicapped hexa­gonal anti­prism (CN = 14) is typical for Zr2 atoms. The Cu atom is enclosed in a eight-vertex polyhedron (octa­hedron with two centered faces). The metallic type of bonding was indicated by an analysis of the inter­atomic distances and electronic structure calculation data.

## Related literature

For general background, see: Dolotko *et al.* (2003 ▶); Giza *et al.* (2001 ▶, 2009 ▶); Zatorska *et al.* (2002*a* ▶,*b* ▶, 2004 ▶). For isotypic structures, see: Garcia & Corbett (1990 ▶); Pöttgen (1997 ▶); Rieger & Parthé (1965 ▶); Stetskiv *et al.* (2011 ▶). For calculation of the electronic structure using the tight-binding linear muffin-tin orbital (TB–LMTO) method in the atomic spheres approximation, see: Andersen (1975 ▶); Andersen & Jepsen (1984 ▶); Andersen *et al.* (1985 ▶, 1986 ▶).

## Experimental

### 

#### Crystal data

Zr_5_CuBi_3_
*M* *_r_* = 1146.58
Hexagonal, 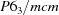

*a* = 8.8712 (4) Å
*c* = 6.0246 (3) Å
*V* = 410.60 (3) Å^3^
*Z* = 2
Mo *K*α radiation
μ = 72.54 mm^−1^
*T* = 293 K
0.08 × 0.04 × 0.02 mm

#### Data collection

Oxford Diffraction Xcalibur3 CCD diffractometer
Absorption correction: analytical (*CrysAlis RED*; Oxford Diffraction, 2008 ▶) *T*_min_ = 0.231, *T*_max_ = 0.654
1713 measured reflections
193 independent reflections
185 reflections with *I* > 2σ(*I*)
*R*_int_ = 0.136

#### Refinement

*R*[*F* ^2^ > 2σ(*F* ^2^)] = 0.023
*wR*(*F* ^2^) = 0.041
*S* = 0.87
193 reflections
13 parameters
Δρ_max_ = 1.92 e Å^−3^
Δρ_min_ = −1.54 e Å^−3^

### 

Data collection: *CrysAlis CCD* (Oxford Diffraction, 2008 ▶); cell refinement: *CrysAlis CCD*; data reduction: *CrysAlis RED* (Oxford Diffraction, 2008 ▶); program(s) used to solve structure: *SHELXS97* (Sheldrick, 2008 ▶); program(s) used to refine structure: *SHELXL97* (Sheldrick, 2008 ▶); molecular graphics: *DIAMOND* (Brandenburg, 2006 ▶); software used to prepare material for publication: *SHELXL97*.

## Supplementary Material

Crystal structure: contains datablock(s) I, New_Global_Publ_Block. DOI: 10.1107/S1600536813019235/ff2112sup1.cif

Structure factors: contains datablock(s) I. DOI: 10.1107/S1600536813019235/ff2112Isup2.hkl

Additional supplementary materials:  crystallographic information; 3D view; checkCIF report

## Figures and Tables

**Fig. 1.**
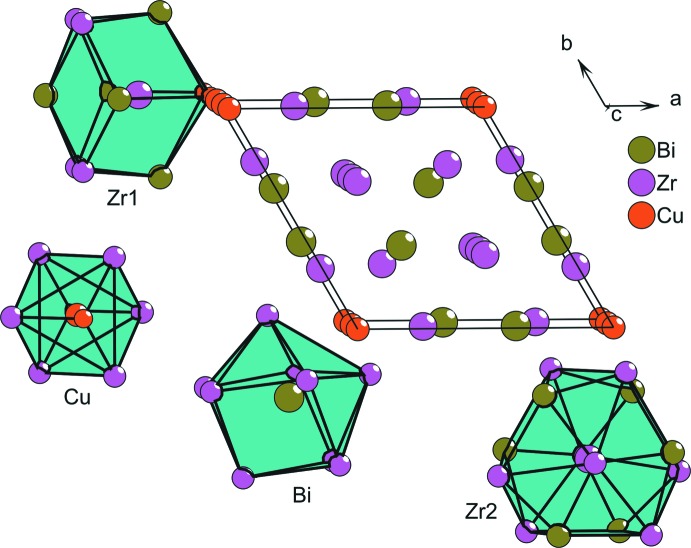
The projection of the unit cell and coordination polyhedra of the atoms.

**Fig. 2.**
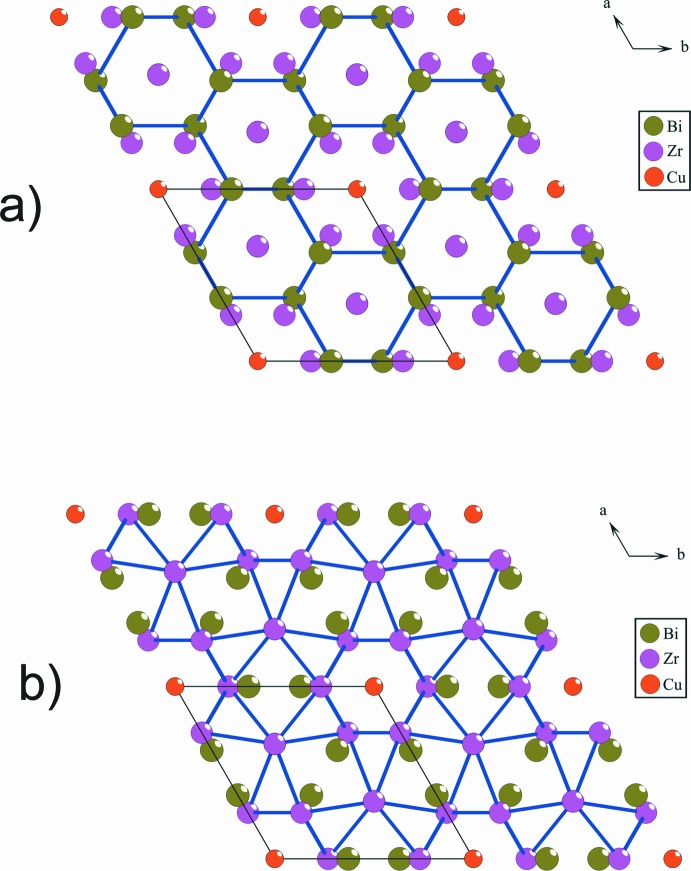
The distribution of Zr and Cu atoms in three-dimensional-nets from Bi atoms (a) and distribution of Bi and Cu atoms in three-dimensional-nets from Zr atoms (b).

**Fig. 3.**
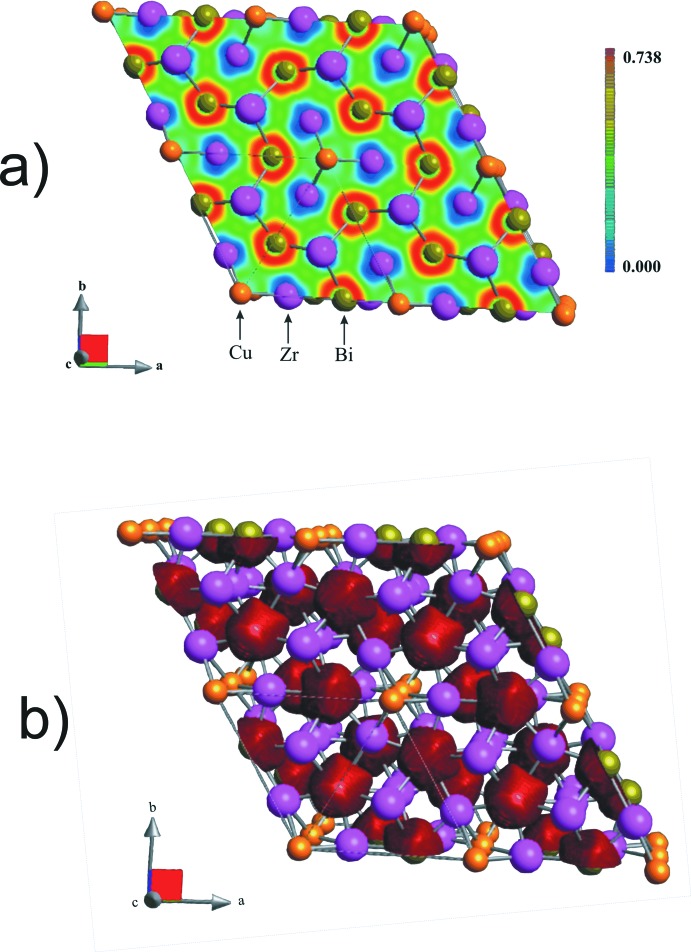
(a) The electron localization function (ELF) mapping and (b) isosurfaces of the electron localization function around the atoms for Zr5CuBi3.

**Fig. 4.**
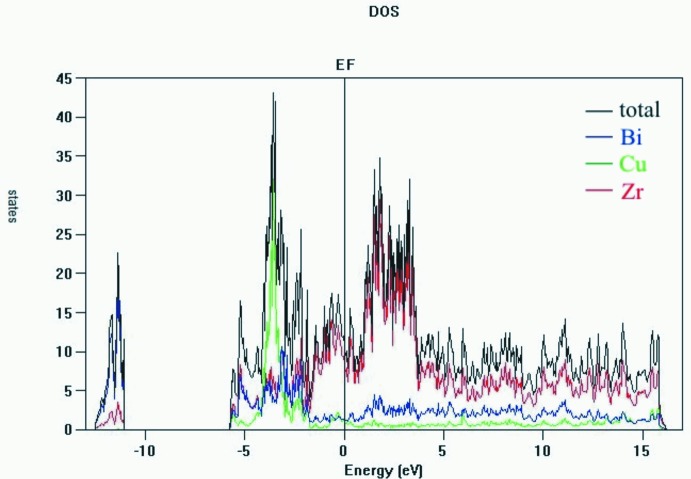
Total and partial DOS (densities of states) for Zr5CuBi3.

## supplementary crystallographic information

### Comment

Zirconium intermetallic compounds are extensively investigated for the last 40 years as possible hydrogen storage materials. The results that we present in this paper is a continuation of the systematic study that we carried out for zirconium alloys with transition metals (Giza *et al.*, 2001; Dolotko *et al.*, 2003) as well as *s*-and *p*-elements (Zatorska *et al.*, 2002*a*,*b*; 2004; Giza *et al.*, 2009). So far, in the literature no data on ternary intermetallic compounds of Zr—Cu—Bi system has been found. However, it is known that closely related systems such as Zr—Cu—Sn (Pöttgen, 1997), Zr—Cu—Pb (Rieger & Parthé, 1965) and Zr—Cu—Sb (Garcia & Corbett, 1990) form Zr_5_Cu*X*_3_ (where *X*=Sn, Pb, Sb) compounds with hexagonal Hf_5_CuSn_3_ structure type (superstructure to Ti_5_Ga_4_-type) with space group *P6_3_/mcm*. Studying alloys of the Zr—Cu—Bi system we found the existence of isostructural Zr_5_CuBi_3_ compound and investigated its structure by single-crystal method. The projection of the unit cell and coordination polyhedra of the atoms are shown in Fig. 1. The environment of the Bi atoms is a tetragonal antiprism with one added atom and a coordination number equal 9. The polyhedron of Zr1 atom is a defective cubooctahedron with a coordination number equal 11. The bicapped hexagonal antiprism (c.n.=14) is typical for Zr2 atom. The Cu atom is enclosed in a 8-vertex polyhedron (octahedron with two centered faces). The distribution of zirconium and copper atoms in three-dimensional-nets consisted of Bi atoms are shown in Fig. 2a and distribution of bismuth and copper atoms in three-dimensional-nets consisted of Zr atoms are shown in Fig. 2b. In the first case the Bi atoms form a 6_3_ corrugated nets and the Zr atoms (second case) form a 3_2_46 nets. The similar atomic nets was described for Tb_5_LiSn_3_ isostructural compound (Stetskiv *et al.*, 2011).

The electronic structure of the Zr_5_CuBi_3_ compound was calculated using the tight-binding linear muffin-tin orbital (TB–LMTO) method in the atomic spheres approximation (TB– LMTO–ASA; Andersen, 1975; Andersen & Jepsen, 1984; Andersen *et al.*, 1985, 1986), using the experimental crystallographic data which are presented here. The Zr and Cu atoms donate their electrons to the Bi atoms. Therefore positive charge density can be observed around the atoms of transition elements (Zr and Cu) and negative charge density is around the bismuth atoms. The electron localization function (ELF) mapping and isosurfaces (ISO) are presented in Fig. 3a and Fig. 3b, respectively. The total and partial densities of states (DOS) of Zr_5_CuBi_3_ compound calculated by the TB–LMTO–ASA method are shown in Fig. 4. The Fermi level (EF) lies in a continuous DOS region indicating a metallic character for the title compound. The metallic type of bonding was confirmed also by an analysis of the interatomic distances.

### Experimental

The title compound was prepared from elemental zirconium (foil, 0.25 mm thick 99.8 at.%, Aldrich), copper (powder, pure, POCH) and bismuth (granules, 99.5 at.%, POCH). The pieces of the pure metals with a stoichiometry Zr_50_Cu_20_Bi_30_ were pressed into pellet. The sample was melted in arc furnace under continuous argon flow. The losses in alloy composition during melting were checked by weight comparison of the initial mixtures and the alloys. Metallic grey prismatic crystals were found in a crushed alloy using a conventional light microscope.

### Refinement

The structure was solved after the analytical absorption correction. In the first stage of the refinement, the positions of the Zr, Cu and Bi atoms were obtained correctly by direct methods. After the last cycle of refinement the highest peak of 1.915 e/Å3 is at (0; 0.4552; 1/4) and 0.76 Å away from the Bi atom. The deepest hole -1.539 e/Å3 is at (0.2424; 0; 1/4) and 1.12 Å away from the same atom.

### Crystal data

**Table:**

Zr_5_CuBi_3_	*D*_x_ = 9.274 Mg m^−^^3^
*M**_r_* = 1146.58	Mo *K*α radiation, λ = 0.71073 Å
Hexagonal, *P*6_3_/*m**c**m*	Cell parameters from 185 reflections
Hall symbol: -P 6c 2	θ = 2.7–27.4°
*a* = 8.8712 (4) Å	µ = 72.54 mm^−^^1^
*c* = 6.0246 (3) Å	*T* = 293 K
*V* = 410.60 (3) Å^3^	Prism, metallic grey
*Z* = 2	0.08 × 0.04 × 0.02 mm
*F*(000) = 956	

### Data collection

**Table:**

Oxford Diffraction Xcalibur3 CCD diffractometer	193 independent reflections
Radiation source: fine-focus sealed tube	185 reflections with *I* > 2σ(*I*)
Graphite monochromator	*R*_int_ = 0.136
Detector resolution: 0 pixels mm^-1^	θ_max_ = 27.4°, θ_min_ = 2.7°
ω scans	*h* = −11→11
Absorption correction: analytical (*CrysAlis RED*; Oxford Diffraction, 2008)	*k* = −11→11
*T*_min_ = 0.231, *T*_max_ = 0.654	*l* = 0→7
1713 measured reflections	

### Refinement

**Table:**

Refinement on *F*^2^	0 restraints
Least-squares matrix: full	Primary atom site location: structure-invariant direct methods
*R*[*F*^2^ > 2σ(*F*^2^)] = 0.023	Secondary atom site location: difference Fourier map
*wR*(*F*^2^) = 0.041	*w* = 1/[σ^2^(*F*_o_^2^) + (0.010*P*)^2^] where *P* = (*F*_o_^2^ + 2*F*_c_^2^)/3
*S* = 0.87	(Δ/σ)_max_ < 0.001
193 reflections	Δρ_max_ = 1.92 e Å^−^^3^
13 parameters	Δρ_min_ = −1.54 e Å^−^^3^

### Special details

**Table:**

Geometry. All e.s.d.'s (except the e.s.d. in the dihedral angle between two l.s. planes) are estimated using the full covariance matrix. The cell e.s.d.'s are taken into account individually in the estimation of e.s.d.'s in distances, angles and torsion angles; correlations between e.s.d.'s in cell parameters are only used when they are defined by crystal symmetry. An approximate (isotropic) treatment of cell e.s.d.'s is used for estimating e.s.d.'s involving l.s. planes.
Refinement. Refinement of *F*^2^ against ALL reflections. The weighted *R*-factor *wR* and goodness of fit *S* are based on *F*^2^, conventional *R*-factors *R* are based on *F*, with *F* set to zero for negative *F*^2^. The threshold expression of *F*^2^ > σ(*F*^2^) is used only for calculating *R*-factors(gt) *etc*. and is not relevant to the choice of reflections for refinement. *R*-factors based on *F*^2^ are statistically about twice as large as those based on *F*, and *R*- factors based on ALL data will be even larger.

### Fractional atomic coordinates and isotropic or equivalent isotropic displacement parameters (Å^2^)

**Table:**

	*x*	*y*	*z*	*U*_iso_*/*U*_eq_	
Bi1	0.63082 (6)	0.63082 (6)	0.2500	0.00715 (19)	
Zr1	0.26831 (17)	0.26831 (17)	0.2500	0.0083 (3)	
Zr2	0.6667	0.3333	0.0000	0.0103 (4)	
Cu1	0.0000	0.0000	0.0000	0.0102 (10)	

### Atomic displacement parameters (Å^2^)

**Table:**

	*U*^11^	*U*^22^	*U*^33^	*U*^12^	*U*^13^	*U*^23^
Bi1	0.0060 (2)	0.0060 (2)	0.0098 (3)	0.0033 (2)	0.000	0.000
Zr1	0.0075 (4)	0.0075 (4)	0.0102 (8)	0.0039 (5)	0.000	0.000
Zr2	0.0132 (7)	0.0132 (7)	0.0045 (11)	0.0066 (3)	0.000	0.000
Cu1	0.0106 (14)	0.0106 (14)	0.009 (3)	0.0053 (7)	0.000	0.000

### Geometric parameters (Å, º)

**Table:**

Bi1—Zr1^i^	2.9319 (6)	Zr2—Zr2^ii^	3.0123 (2)
Bi1—Zr1^ii^	2.9319 (6)	Zr2—Zr2^viii^	3.0123 (2)
Bi1—Zr1^iii^	3.1424 (5)	Zr2—Bi1^ix^	3.1896 (2)
Bi1—Zr1^iv^	3.1424 (5)	Zr2—Bi1^ii^	3.1896 (2)
Bi1—Zr2^v^	3.1896 (2)	Zr2—Bi1^vi^	3.1896 (2)
Bi1—Zr2^ii^	3.1896 (2)	Zr2—Bi1^x^	3.1896 (2)
Bi1—Zr2	3.1896 (2)	Zr2—Bi1^iv^	3.1896 (2)
Bi1—Zr2^iv^	3.1896 (2)	Zr2—Zr1^ii^	3.6126 (9)
Bi1—Zr1	3.2159 (17)	Zr2—Zr1^vi^	3.6126 (9)
Zr1—Cu1	2.8167 (13)	Zr2—Zr1^ix^	3.6126 (9)
Zr1—Cu1^v^	2.8167 (13)	Zr2—Zr1^x^	3.6126 (9)
Zr1—Bi1^vi^	2.9319 (6)	Cu1—Zr1^xi^	2.8167 (13)
Zr1—Bi1^vii^	2.9319 (6)	Cu1—Zr1^ix^	2.8167 (13)
Zr1—Bi1^iii^	3.1424 (5)	Cu1—Zr1^xii^	2.8167 (13)
Zr1—Bi1^iv^	3.1424 (5)	Cu1—Zr1^xiii^	2.8167 (13)
Zr1—Zr2^ii^	3.6126 (9)	Cu1—Zr1^xiv^	2.8167 (13)
Zr1—Zr2^v^	3.6126 (9)	Cu1—Cu1^v^	3.0123 (2)
Zr1—Zr2	3.6126 (9)	Cu1—Cu1^xii^	3.0123 (2)
Zr1—Zr2^iv^	3.6126 (9)		
			
Zr1^i^—Bi1—Zr1^ii^	89.35 (6)	Zr2^viii^—Zr2—Bi1^ix^	61.822 (2)
Zr1^i^—Bi1—Zr1^iii^	78.32 (3)	Zr2^ii^—Zr2—Bi1^ii^	61.822 (2)
Zr1^ii^—Bi1—Zr1^iii^	78.32 (3)	Zr2^viii^—Zr2—Bi1^ii^	118.178 (2)
Zr1^i^—Bi1—Zr1^iv^	78.32 (3)	Bi1^ix^—Zr2—Bi1^ii^	88.460 (16)
Zr1^ii^—Bi1—Zr1^iv^	78.32 (3)	Zr2^ii^—Zr2—Bi1^vi^	61.822 (2)
Zr1^iii^—Bi1—Zr1^iv^	146.91 (6)	Zr2^viii^—Zr2—Bi1^vi^	118.178 (2)
Zr1^i^—Bi1—Zr2^v^	72.20 (3)	Bi1^ix^—Zr2—Bi1^vi^	73.185 (10)
Zr1^ii^—Bi1—Zr2^v^	145.409 (19)	Bi1^ii^—Zr2—Bi1^vi^	99.528 (3)
Zr1^iii^—Bi1—Zr2^v^	69.571 (14)	Zr2^ii^—Zr2—Bi1^x^	118.178 (2)
Zr1^iv^—Bi1—Zr2^v^	123.798 (7)	Zr2^viii^—Zr2—Bi1^x^	61.822 (2)
Zr1^i^—Bi1—Zr2^ii^	145.409 (19)	Bi1^ix^—Zr2—Bi1^x^	99.528 (3)
Zr1^ii^—Bi1—Zr2^ii^	72.20 (3)	Bi1^ii^—Zr2—Bi1^x^	73.185 (10)
Zr1^iii^—Bi1—Zr2^ii^	69.571 (14)	Bi1^vi^—Zr2—Bi1^x^	170.093 (17)
Zr1^iv^—Bi1—Zr2^ii^	123.798 (7)	Zr2^ii^—Zr2—Bi1^iv^	118.178 (2)
Zr2^v^—Bi1—Zr2^ii^	106.815 (9)	Zr2^viii^—Zr2—Bi1^iv^	61.822 (2)
Zr1^i^—Bi1—Zr2	145.409 (19)	Bi1^ix^—Zr2—Bi1^iv^	99.528 (3)
Zr1^ii^—Bi1—Zr2	72.20 (3)	Bi1^ii^—Zr2—Bi1^iv^	170.093 (17)
Zr1^iii^—Bi1—Zr2	123.798 (7)	Bi1^vi^—Zr2—Bi1^iv^	88.460 (16)
Zr1^iv^—Bi1—Zr2	69.571 (14)	Bi1^x^—Zr2—Bi1^iv^	99.528 (3)
Zr2^v^—Bi1—Zr2	137.327 (18)	Zr2^ii^—Zr2—Bi1	61.822 (2)
Zr2^ii^—Bi1—Zr2	56.356 (5)	Zr2^viii^—Zr2—Bi1	118.178 (2)
Zr1^i^—Bi1—Zr2^iv^	72.20 (3)	Bi1^ix^—Zr2—Bi1	170.093 (17)
Zr1^ii^—Bi1—Zr2^iv^	145.409 (19)	Bi1^ii^—Zr2—Bi1	99.528 (3)
Zr1^iii^—Bi1—Zr2^iv^	123.798 (7)	Bi1^vi^—Zr2—Bi1	99.528 (3)
Zr1^iv^—Bi1—Zr2^iv^	69.571 (14)	Bi1^x^—Zr2—Bi1	88.460 (16)
Zr2^v^—Bi1—Zr2^iv^	56.356 (5)	Bi1^iv^—Zr2—Bi1	73.185 (9)
Zr2^ii^—Bi1—Zr2^iv^	137.327 (18)	Zr2^ii^—Zr2—Zr1^ii^	65.360 (7)
Zr2—Bi1—Zr2^iv^	106.815 (10)	Zr2^viii^—Zr2—Zr1^ii^	114.640 (7)
Zr1^i^—Bi1—Zr1	135.33 (3)	Bi1^ix^—Zr2—Zr1^ii^	139.216 (18)
Zr1^ii^—Bi1—Zr1	135.33 (3)	Bi1^ii^—Zr2—Zr1^ii^	56.01 (2)
Zr1^iii^—Bi1—Zr1	106.55 (3)	Bi1^vi^—Zr2—Zr1^ii^	127.097 (6)
Zr1^iv^—Bi1—Zr1	106.55 (3)	Bi1^x^—Zr2—Zr1^ii^	54.600 (5)
Zr2^v^—Bi1—Zr1	68.664 (9)	Bi1^iv^—Zr2—Zr1^ii^	114.38 (2)
Zr2^ii^—Bi1—Zr1	68.664 (9)	Bi1—Zr2—Zr1^ii^	50.598 (19)
Zr2—Bi1—Zr1	68.664 (9)	Zr2^ii^—Zr2—Zr1^vi^	65.360 (7)
Zr2^iv^—Bi1—Zr1	68.664 (9)	Zr2^viii^—Zr2—Zr1^vi^	114.640 (7)
Cu1—Zr1—Cu1^v^	64.65 (3)	Bi1^ix^—Zr2—Zr1^vi^	54.600 (5)
Cu1—Zr1—Bi1^vi^	77.64 (3)	Bi1^ii^—Zr2—Zr1^vi^	50.598 (19)
Cu1^v^—Zr1—Bi1^vi^	77.64 (3)	Bi1^vi^—Zr2—Zr1^vi^	56.01 (2)
Cu1—Zr1—Bi1^vii^	77.64 (3)	Bi1^x^—Zr2—Zr1^vi^	114.38 (2)
Cu1^v^—Zr1—Bi1^vii^	77.64 (3)	Bi1^iv^—Zr2—Zr1^vi^	139.216 (18)
Bi1^vi^—Zr1—Bi1^vii^	150.65 (6)	Bi1—Zr2—Zr1^vi^	127.097 (6)
Cu1—Zr1—Bi1^iii^	138.87 (4)	Zr1^ii^—Zr2—Zr1^vi^	103.844 (8)
Cu1^v^—Zr1—Bi1^iii^	74.221 (13)	Zr2^ii^—Zr2—Zr1^ix^	114.640 (7)
Bi1^vi^—Zr1—Bi1^iii^	94.137 (2)	Zr2^viii^—Zr2—Zr1^ix^	65.360 (7)
Bi1^vii^—Zr1—Bi1^iii^	94.137 (2)	Bi1^ix^—Zr2—Zr1^ix^	56.01 (2)
Cu1—Zr1—Bi1^iv^	74.221 (13)	Bi1^ii^—Zr2—Zr1^ix^	139.216 (18)
Cu1^v^—Zr1—Bi1^iv^	138.87 (4)	Bi1^vi^—Zr2—Zr1^ix^	54.600 (5)
Bi1^vi^—Zr1—Bi1^iv^	94.137 (2)	Bi1^x^—Zr2—Zr1^ix^	127.097 (6)
Bi1^vii^—Zr1—Bi1^iv^	94.137 (2)	Bi1^iv^—Zr2—Zr1^ix^	50.598 (19)
Bi1^iii^—Zr1—Bi1^iv^	146.91 (6)	Bi1—Zr2—Zr1^ix^	114.38 (2)
Cu1—Zr1—Bi1	147.675 (17)	Zr1^ii^—Zr2—Zr1^ix^	164.10 (4)
Cu1^v^—Zr1—Bi1	147.675 (17)	Zr1^vi^—Zr2—Zr1^ix^	89.71 (3)
Bi1^vi^—Zr1—Bi1	104.67 (3)	Zr2^ii^—Zr2—Zr1^x^	114.640 (7)
Bi1^vii^—Zr1—Bi1	104.67 (3)	Zr2^viii^—Zr2—Zr1^x^	65.360 (7)
Bi1^iii^—Zr1—Bi1	73.45 (3)	Bi1^ix^—Zr2—Zr1^x^	50.598 (19)
Bi1^iv^—Zr1—Bi1	73.45 (3)	Bi1^ii^—Zr2—Zr1^x^	54.600 (5)
Cu1—Zr1—Zr2^ii^	134.725 (16)	Bi1^vi^—Zr2—Zr1^x^	114.38 (2)
Cu1^v^—Zr1—Zr2^ii^	104.942 (7)	Bi1^x^—Zr2—Zr1^x^	56.01 (2)
Bi1^vi^—Zr1—Zr2^ii^	57.205 (10)	Bi1^iv^—Zr2—Zr1^x^	127.097 (6)
Bi1^vii^—Zr1—Zr2^ii^	146.09 (3)	Bi1—Zr2—Zr1^x^	139.216 (18)
Bi1^iii^—Zr1—Zr2^ii^	55.829 (13)	Zr1^ii^—Zr2—Zr1^x^	89.71 (3)
Bi1^iv^—Zr1—Zr2^ii^	103.75 (3)	Zr1^vi^—Zr2—Zr1^x^	64.19 (4)
Bi1—Zr1—Zr2^ii^	55.32 (2)	Zr1^ix^—Zr2—Zr1^x^	103.844 (8)
Cu1—Zr1—Zr2^v^	134.725 (16)	Zr1—Cu1—Zr1^xi^	180.00 (6)
Cu1^v^—Zr1—Zr2^v^	104.942 (7)	Zr1—Cu1—Zr1^ix^	85.92 (2)
Bi1^vi^—Zr1—Zr2^v^	146.09 (3)	Zr1^xi^—Cu1—Zr1^ix^	94.08 (2)
Bi1^vii^—Zr1—Zr2^v^	57.205 (11)	Zr1—Cu1—Zr1^xii^	85.92 (2)
Bi1^iii^—Zr1—Zr2^v^	55.829 (13)	Zr1^xi^—Cu1—Zr1^xii^	94.08 (2)
Bi1^iv^—Zr1—Zr2^v^	103.75 (3)	Zr1^ix^—Cu1—Zr1^xii^	94.08 (2)
Bi1—Zr1—Zr2^v^	55.32 (2)	Zr1—Cu1—Zr1^xiii^	94.08 (2)
Zr2^ii^—Zr1—Zr2^v^	90.29 (3)	Zr1^xi^—Cu1—Zr1^xiii^	85.92 (2)
Cu1—Zr1—Zr2	104.942 (7)	Zr1^ix^—Cu1—Zr1^xiii^	85.92 (2)
Cu1^v^—Zr1—Zr2	134.725 (16)	Zr1^xii^—Cu1—Zr1^xiii^	180.00 (3)
Bi1^vi^—Zr1—Zr2	57.205 (10)	Zr1—Cu1—Zr1^xiv^	94.08 (2)
Bi1^vii^—Zr1—Zr2	146.09 (3)	Zr1^xi^—Cu1—Zr1^xiv^	85.92 (2)
Bi1^iii^—Zr1—Zr2	103.75 (3)	Zr1^ix^—Cu1—Zr1^xiv^	180.00 (3)
Bi1^iv^—Zr1—Zr2	55.829 (13)	Zr1^xii^—Cu1—Zr1^xiv^	85.92 (2)
Bi1—Zr1—Zr2	55.32 (2)	Zr1^xiii^—Cu1—Zr1^xiv^	94.08 (2)
Zr2^ii^—Zr1—Zr2	49.279 (13)	Zr1—Cu1—Cu1^v^	57.675 (17)
Zr2^v^—Zr1—Zr2	110.65 (4)	Zr1^xi^—Cu1—Cu1^v^	122.325 (17)
Cu1—Zr1—Zr2^iv^	104.942 (7)	Zr1^ix^—Cu1—Cu1^v^	122.325 (17)
Cu1^v^—Zr1—Zr2^iv^	134.725 (16)	Zr1^xii^—Cu1—Cu1^v^	122.325 (17)
Bi1^vi^—Zr1—Zr2^iv^	146.09 (3)	Zr1^xiii^—Cu1—Cu1^v^	57.675 (17)
Bi1^vii^—Zr1—Zr2^iv^	57.205 (10)	Zr1^xiv^—Cu1—Cu1^v^	57.675 (17)
Bi1^iii^—Zr1—Zr2^iv^	103.75 (3)	Zr1—Cu1—Cu1^xii^	122.325 (17)
Bi1^iv^—Zr1—Zr2^iv^	55.829 (13)	Zr1^xi^—Cu1—Cu1^xii^	57.675 (17)
Bi1—Zr1—Zr2^iv^	55.32 (2)	Zr1^ix^—Cu1—Cu1^xii^	57.675 (17)
Zr2^ii^—Zr1—Zr2^iv^	110.65 (4)	Zr1^xii^—Cu1—Cu1^xii^	57.675 (17)
Zr2^v^—Zr1—Zr2^iv^	49.279 (13)	Zr1^xiii^—Cu1—Cu1^xii^	122.325 (17)
Zr2—Zr1—Zr2^iv^	90.29 (3)	Zr1^xiv^—Cu1—Cu1^xii^	122.325 (17)
Zr2^ii^—Zr2—Zr2^viii^	180.0	Cu1^v^—Cu1—Cu1^xii^	180.0
Zr2^ii^—Zr2—Bi1^ix^	118.178 (2)		

Symmetry codes: (i) −*y*+1, *x*−*y*+1, *z*; (ii) −*x*+*y*+1, −*x*+1, −*z*+1/2; (iii) −*x*+1, −*y*+1, −*z*+1; (iv) −*x*+1, −*y*+1, −*z*; (v) *x*−*y*, *x*, *z*+1/2; (vi) −*y*+1, *x*−*y*, *z*; (vii) −*x*+*y*, −*x*+1, −*z*+1/2; (viii) −*x*+*y*+1, −*x*+1, −*z*−1/2; (ix) *y*, −*x*+*y*, −*z*; (x) *x*−*y*+1, *x*, *z*−1/2; (xi) −*x*, −*y*, −*z*; (xii) *x*−*y*, *x*, *z*−1/2; (xiii) −*x*+*y*, −*x*, −*z*+1/2; (xiv) −*y*, *x*−*y*, *z*.
